# Recommendation for the diagnosis and management of immune checkpoint inhibitor related infections

**DOI:** 10.1111/1759-7714.13313

**Published:** 2020-01-22

**Authors:** Minya Lu, Li Zhang, Yue Li, Hanping Wang, Xiaoxiao Guo, Jiaxin Zhou, Lian Duan, Xiaoyan Si, Yingchun Xu, Li Zhang

**Affiliations:** ^1^ Department of Laboratory Medicine, Peking Union Medical College Hospital Chinese Academy of Medical Sciences Beijing China; ^2^ Beijing Key Laboratory for Mechanisms Research and Precision Diagnosis of Invasive Fungal Diseases (BZ0447) Beijing China; ^3^ Department of Gastroenterology, Peking Union Medical College Hospital Chinese Academy of Medical Sciences Beijing China; ^4^ Department of Respirology, Peking Union Medical College Hospital Chinese Academy of Medical Sciences Beijing China; ^5^ Department of Cardiology, Peking Union Medical College Hospital Chinese Academy of Medical Sciences Beijing China; ^6^ Department of Rheumatology and Immunology, Peking Union Medical College Hospital Chinese Academy of Medical Sciences Beijing China; ^7^ Department of Endocrinology, Peking Union Medical College Hospital Chinese Academy of Medical Sciences Beijing China

**Keywords:** Immune checkpoint, immune‐related adverse events, infections, PD‐1/PD‐L1 inhibitors

## Abstract

Immune checkpoint inhibitors (ICIs) have been widely used in the management of malignant tumors. Programmed death 1 (PD‐1)/PD‐1 ligand (PD‐L1) inhibitors have been introduced to treat non‐small cell lung cancer (NSCLC) in recent years. Currently, PD‐1/PD‐L1 inhibitors are considered to have minor side effects and do not independently increase the risk of infection. However, they may cause immune‐related adverse events (irAEs) that require immunosuppressive therapy with corticosteroids and/or immunosuppressants, leading to opportunistic infections. Furthermore, there have been reports describing reactivation of chronic/latent infections without irAEs or having received immunosuppressants. Thus, immune checkpoint inhibitor related infections have received more attention worldwide. In this paper, we review available clinical data, describe the potential mechanism, and propose recommendations for the diagnosis and clinical management of PD‐1/PD‐L1 inhibitor‐related infections.

## Introduction

In recent years, programmed death 1 (PD‐1)/PD‐1 ligand (PD‐L1) inhibitors have been used in the treatment of non‐small cell lung cancer (NSCLC). Immunotherapy alone or in combination with chemotherapy has been recommended as initial therapy for advanced NSCLC without EGFR, ALK or ROS1 mutation.^1^

Treatment with PD‐1/PD‐L1 inhibitors are generally considered to result in minor side effects. It is currently believed that PD‐1/PD‐L1 inhibitors do not increase the risk of infection because they promote T‐cell effector functions. However, immune‐related adverse events (irAEs) induced by PD‐1/PD‐L1 inhibitors may require treatment with immunosuppressive agents, which could cause opportunistic infections.^2^, ^3^ In addition, there have been several reports describing reactivation of latent/chronic infections during immunotherapy without irAEs or having received immunosuppressants.^4^

## Mechanism of action and indications

PD‐1 is a key immune checkpoint receptor that inhibits T‐cell activity and is primarily expressed on activated CD8+ and CD4+ T cells.^5^, ^6^ Its inhibitory function is mediated primarily in peripheral tissues by engaging with PD‐1 ligands (PD‐L1 and PD‐L2). PD‐L1 expressed on the surface of tumor cells and cells in the tumor microenvironment can be upregulated by interferon γ (IFN‐γ) secreted by T cells. PD‐1 engages with upregulated PD‐L1 and subsequently inhibits T cell function. Blockage of PD‐1/PD‐L1 can thus enhance T cell activity and restore antitumor immunity.^7^ In clinical practice, PD‐1/PD‐L1 expression intensity has been shown to be associated with the clinical benefit in various tumor types including as NSCLC^8^ and melanoma.^9^ In recent years, PD‐1 inhibitors such as pembrolizumab and nivolumab, as well as PD‐L1 inhibitor atezolizumab have been approved for the treatment of several tumor types including NSCLC.

## Clinical data review and description of potential mechanism of infections

For patients receiving PD‐1/PD‐L1 inhibitors, current large randomized clinical trials have not shown any increased risk of infection.^10^, ^11^, ^12^, ^13^, ^14^, ^15^, ^16^ However, patients may require immunosuppressants such as corticosteroids, TNF‐α targeted agents when irAEs occur, possibly leading to opportunistic infections.

A study by Del Castillo *et al*. retrospectively analyzed melanoma patients receiving immune checkpoint inhibitors in a tertiary care cancer center. A total of 898 courses were analyzed, including 658 treated with ipilimumab (CTLA‐4 inhibitor), 52 with nivolumab, 83 with pembrolizumab and 80 with nivolumab combined with ipilimumab. Among patients receiving PD‐1 inhibitor monotherapy or combined therapy, 13 (6.0%) episodes of severe infections had occurred, mostly in patients treated with both nivolumab and ipilimumab. The most common pathogen was bacteria, followed by fungi (including two cases of pneumocystis infection) and virus. The main risk factors for infection were receipt of corticosteroids and/or infliximab (TNF‐α targeted agent).^3^ Another study of 167 NSCLC patients treated with nivolumab reported that 33 infections occurred in total, of which 25 were bacterial, two were fungal and six were viral. Diabetes mellitus was an independent risk factor for infection.^2^

Of note, among patients without irAEs or additional immunosuppressive therapy, there exists a potential risk of reactivation of chronic/latent infections. Seven cases have been recently reported that describe reactivation of latent tuberculosis infection (LTBI), most occurring within three months after treatment with PD‐1/PD‐L1 inhibitors.^4^, ^17^, ^18^, ^19^ The possible mechanism may involve a boost of T helper cell (TH)1 function,^17^ resembling the immune reconstitution inflammatory syndrome (IRIS) observed in HIV patients at the beginning of antiretroviral therapy. According to REISAMIC (a French, multicenter, prospective registry), the relative incidence of tuberculosis (TB) was approximately one in 1000 among cancer patients receiving PD1/PD‐L1 inhibitors.^20^ Furthermore, in 2018, Japan reported a case of exacerbation of chronic progressive pulmonary aspergillosis (CPPA) in a patient receiving 20 courses of nivolumab.^21^ The same year, another patient treated with nivolumab was reported to have developed varicella zoster virus (VZV) infection during treatment.^22^ None of the aforementioned cases had irAEs or immunosuppressive therapy.

Conversely, several studies have shown that enhancement of the T cell effect by PD‐1/PD‐L1 blockage may be beneficial to enhancing pathogen clearance and improving survival among sepsis patients and immunodeficiency hosts.^23^, ^24^, ^25^ A case has been previously reported in which a patient with invasive mucormycosis was successfully treated with nivolumab combined with IFN‐γ.^26^ Therefore, further studies are needed to investigate the relationship between PD‐1/PD‐L1 blockage and infection. Table 1 provides a summary of PD‐1/PD‐L1 inhibitor related infections.

**Table 1:** Summary of PD‐1/PD‐L1 inhibitor related infections

Type	Possible mechanism	Risk factors	Common pathogens
Opportunistic infections related to irAEs	IrAEs required corticosteroids and/or immunosuppressants, leading to temporary immunesuppression	Use of corticosteroids and/or TNF‐α inhibitors；Diabetes	Opportunistic infections caused by bacteria, fungi, virus *et al*.
Reactivation of chronic/latent infections	Resembling the IRIS; Boosting TH1 function^17^	Unknown	LTBI (7) CPPA (1) VZV (1)

CPPA, chronic progressive pulmonary aspergillosis; irAEs, immune‐related adverse event; IRIS, immune reconstitution inflammatory syndrome; LTBI, latent tuberculosis infection; VZV, varicella zoster virus.

## Diagnosis

### Pretreatment monitoring

Prior to initiation of immunotherapy, screening for latent/chronic infections is advisable, as immunosuppressors may be required during treatment.

- Bacterial infections should be closely monitored during immune checkpoint inhibitor treatment. For patients who have already developed severe or opportunistic infections, caution should be taken before commencing treatment.
- To detect LTBI, screening tests including the tuberculin skin test (TST) or interferon‐gamma release assays (IGRAs), are advisable for patients who may require immunotherapy. For patients who have positive screening test results, chest imaging may be performed. Further evaluation for TB may be carried out for patients with abnormal chest imaging results.
- Recent studies indicate that PD‐1/PD‐L1 inhibitors do not show increased toxicity in patients with tumor in the context of hepatitis B virus (HBV) or hepatitis C virus (HCV) infection.^27^, ^28^ However, for patients developing irAEs, treatment with immunosuppressive therapy may increase the risk of reactivation of HBV/HCV infection. Furthermore, for patients without irAEs or treatment with immunosuppressants, there is a possibility of reactivation of latent/chronic infections following immune checkpoint inhibitor therapy. Thus, screening for HBV/HCV infection may be practical before initiating immunotherapy.
- Similar to HBV/HCV infection, studies have shown no increased toxicity of PD‐1/PD‐L1 inhibitors among patients with tumor and HIV.^29^ However, screening for HIV infection before treatment initiation is advisable because of the underlying possibility of reactivation of latent/chronic infections.
- For patients suspected of having chronic pulmonary aspergillosis, a chest CT scan should be performed.

### Monitoring during and after treatment

- For patients with irAEs who require immunosuppressive therapy such as corticosteroids and/or TNF‐α targeted agents, close monitoring should be maintained to detect early signs of infection. A multidisciplinary approach involving oncologists and infectious disease specialists is highly recommended.^7^
- For HBV/HCV carriers with irAEs receiving TNF‐α targeted agents, close monitoring is needed during and for several months after therapy.^30^
- For patients with latent/chronic infections, close monitoring for developing infection is needed during treatment.

## Treatments

### Infections related to irAEs

- Pneumonia is the most common infection in patients with irAEs.^2^ For patients with signs of infection, initiation of empirical antimicrobial treatment as soon as possible is advised, while further microbiological examinations are needed to identify pathogens. Compound sulfamethoxazole is recommended for PCP treatment, while alternative regimens may be used for patients with sulfa allergy.
- Studies have shown that diabetes is an important risk factor for PD‐1/PD‐L1 inhibitor‐related infections.^2^ Thus, tight glycemic control is recommended during treatment.
- Precautionary measures:
  - For patients with irAEs who are expected to receive prednisone (≥ 20 mg/day) or other equivalent glucocorticoids for at least four weeks, anti‐pneumocystis prophylaxis is recommended.^7^, ^31^
  - For patients with irAEs who are expected to receive prednisone (≥ 20 mg/day) or other equivalent glucocorticoids for at least six weeks, antifungal prophylaxis is recommended.^30^
  - It is recommended to administer prophylaxis against herpes zoster reactivation.^30^
  - Antibiotics for prophylaxis are not advisable as several studies have shown worse outcomes when antibiotics were given to NSCLC patients taking immunotherapy.^32^, ^33^
  - Routine vaccination is recommended by the ESGICH consensus document on the safety of targeted and biological therapies.^7^ However, some studies express concern about the risk of infections when using attenuated live vaccines.^31^ Study results regarding inactivated vaccines are controversial. One study reported increased frequency of irAEs after treatment with inactivated influenza vaccines in cancer patients treated during PD‐1 blockage.^34^ On the contrary, another study suggested that it was safe to use inactivated vaccines for patients receiving PD‐1/PD‐L1 inhibitors.^35^ Therefore, potential risks and benefits should be carefully evaluated before vaccination.
  - Combined use of ipilimumab and PD‐1 inhibitors was associated with a higher rate of irAEs compared with ipilimumab or PD‐1 inhibitor monotherapy.^9^, ^36^, ^37^

### Reactivation of chronic/latent infections

- Rigid glycemic control is recommended during treatment.
- During treatment, close monitoring for developing infection is needed.
- For patients with LTBI reactivation during PD‐1/PD‐L1 blockage, anti‐TB therapy is recommended. The regimen and course of anti‐TB therapy remains inconclusive, considering that there is still a lack of clinical practice on LTBI reactivation during immunotherapy. Most of the existing cases report the use of standard HRZE regimens for initial therapy, while several reports have used two‐, three‐ or five‐drug regimens.^4^, ^17^
  During anti‐TB therapy, liver function should be closely monitored for early detection of anti‐TB drug‐related liver damage to differentiate from PD‐1/PD‐L1 related liver toxicity.^7^, ^31^
- Is it necessary to stop immunotherapy?
  At present, there is still limited data regarding the treatment of active TB during immunotherapy. In general, it is advised to discontinue immune checkpoint blockage temporarily in the presence of active TB, although the timing for resuming immunotherapy is unclear.^38^ However, several cases have been described in which the successful treatment of LTBI reactivation occurred in the course of immunotherapy without discontinuation of immune checkpoint inhibitors.^4^
- For patients with active tuberculosis, anti‐TB therapy is recommended before initiating immune checkpoint blockage.^38^ For patients with latent or suspected tuberculosis, no case has been reported that addresses anti‐TB therapy before immunotherapy.

## Disclosure

The authors have no potential conflicts of interest to disclose.
